# Clinical Significance of *LINC00261* in the Pathogenesis of Pancreatic, Colorectal, Hepatocellular, and Gallbladder Cancer

**DOI:** 10.3390/diseases13030089

**Published:** 2025-03-20

**Authors:** Sanjana Bana, Sia Daffara, Aastha Dagar, Ashutosh Kumar Tiwari, Kanupriya Medhi, Sagarika Mukherjee, Vivek Uttam, Md Rizwan Ansari, Hardeep Singh Tuli, Vikas Yadav, Aklank Jain

**Affiliations:** 1 Non-Coding RNA and Cancer Biology Lab, Department of Zoology, Central University of Punjab, Ghudda, Bathinda 151401, Punjab, India; sanjanabana@gmail.com (S.B.); siadaffara@gmail.com (S.D.); aasthadagar2000@gmail.com (A.D.); ashutoshlokesh@gmail.com (A.K.T.); priyamedhi14@gmail.com (K.M.); sagarikamukherjee444@gmail.com (S.M.); vivekuttam52@gmail.com (V.U.); 2 GD Research Center, 3rd Floor, Jyoti Pinnacle Building, Survey No.11, Kondapur Village, Serilingampally Mandal, Ranga Reddy District, Hyderabad 500081, Telangana, India; rizwanindia@gmail.com; 3 Department of Bio-Sciences and Technology, Maharishi Markandeshwar (Deemed to be University), Mullana, Ambala 133207, Haryana, India; hardeep.biotech@gmail.com; 4 Department of Translational Medicine, Clinical Research Centre, Skåne University Hospital, Lund University, 20213 Malmö, Sweden

**Keywords:** lncRNA, *LINC00261*, pancreatic cancer, hepatocellular carcinoma, colorectal cancer, gall bladder cancer

## Abstract

Pancreatic (PC), colorectal (CRC), hepatocellular (HCC), and gallbladder (GC) cancers together account for nearly 20% of all cancer cases. However, specific biomarkers and therapeutic targets for these cancers are lacking. Diagnosing these cancers early and providing timely, appropriate treatment to improve patient outcomes is crucial. In this context, previous studies, including ours, have highlighted the potential of non-coding RNAs, particularly long non-coding RNAs (lncRNAs), in diagnosing and prognosis of various cancers. This review focuses on the mechanistic role of the recently identified lncRNA *LINC00261* in PC, CRC, HCC, and GC. Our comprehensive literature analysis revealed that *LINC00261* functions as a tumor suppressor, and its reduced expression is associated with larger tumor size, advanced tumor-node-metastasis (TNM) stages, lymphatic metastasis, and poorer overall survival rates. Additionally, we discovered that *LINC00261* acts as a molecular sponge for miRNAs, such as miR-550a-3p, miR-23a-3p, miR-148a, miR-324-3p, and miR-105-5p, regulating critical cancer-related signaling pathways, including PI3K/Akt/mTOR, Protein kinase B, and Mammalian target of rapamycin (mTOR). Further bioinformatic analysis revealed that *LINC00261* regulates key cellular processes, such as protein-DNA complex formation, ribonuclease complex activity, histone deacetylase complexes, and nuclear matrix interactions. Overall, we believe that *LINC00261* holds significant promise as a future biomarker and, when combined with existing treatment strategies, may enhance cancer patient care and survival.

## 1. Introduction

Cancer is one of the most significant global health challenges, impacting individuals and families both emotionally and financially. The gravity of this global issue is highlighted by the latest GLOBOCAN (Global Cancer Observatory) 2022 data, which indicate that cancer incidence reached 196.9 cases per 100,000 individuals [1]. Notably, the global age-standardized rate (ASR) for hepatocellular, pancreatic, colorectal, and gallbladder cancer mortality rates are 7.8% (liver), and 4.8%, 9.3%, and 0.9% for both sexes, respectively [2]. This alarming statistic underscores the urgent need for intensified research and improved treatment options. The high mortality rates associated with hepatocellular, gallbladder, and pancreatic cancers can be attributed mainly to their multifactorial nature, often characterized by uncontrolled cell growth, invasion, and metastasis to distant tissues. Factors such as a poor diet, smoking, pollution, and an aging population significantly contribute to the rising global incidence and mortality rates of cancer [3]. Limited access to early detection and treatment, particularly in low and middle-income countries, also results in higher mortality. Furthermore, projections indicate that over the next 20 years, the death rate may rise substantially due to the absence of early symptoms, late-stage diagnoses, and resistance to current regimen treatments [4].

Recent evidence indicates that long non-coding RNAs having a size of more than 200 nucleotides modulate several biological activities such as epigenetics, immune response regulation, transcription, splicing, translation, cell integrity, migration, cell cycle, and cell proliferation [5,6,7]. Additionally, it has been shown that lncRNAs have a role in the regulation of the critical hallmarks of cancer, including persistent proliferative signaling, evasion of growth suppressors, anti-apoptotic behavior, immune escape, oncogenic inflammation, disruptions in cellular metabolism, and genomic instability [5,8,9]. Recently, we and other researchers have shown the critical role that non-coding RNAs like miRNAs and lncRNAs play in the pathophysiology of many types of cancer [10,11,12,13,14]. Hence, due to their excellent association with the tumorigenesis process, they are suggested as diagnostic/prognostic biomarkers and targets for therapeutic intervention in cancer [15]. Moreover, lncRNA expression was also found to be affected by various clinicopathological features, including Ki-67 levels (Antigen Kiel 67), age, gender, tumor-node metastatic stage, histological grade, and lymph node metastasis age [16].

The long intergenic non-protein coding RNA *LINC00261* has been newly identified as having diverse functions in cancers. *LINC00261* is located at chromosome 20p11.21 (GRCh38/hg38), having Size ≈ 44,299 bases [17,18]. There are some other aliases for the *LINC00261*, such as *TCONS_00027846* (*Transcript consensus number*), *DEANR1* (*Definitive endoderm-associated lncRNA1*), *Onco-lncRNA-17*, *ALIEN* (*A Long Intergenic Non-coding RNA enhancing Neuroblastoma*), and *FALCOR* (*Folate associated long non-coding RNA*) [19]. It functions as a tumor suppressor in thyroid cancer by negatively regulating *EBF1* (*Early B cell factor 1*), which has a partial role in the inhibition of tumor growth and metastasis, and its downregulation is associated with advanced disease and poor prognosis [20]. Moreover, it suppresses viability, migration, and invasion of breast cancer stem cells (BCSC) by sponging the miR-550a-3p to upregulate (SDPR) serum deprivation response protein [21]. *LINC00261* is suppressed by hypermethylation, which causes platinum resistance and a poor prognosis in epithelial ovarian cancer (EOC) through the miR-545-3p/MT1M (metallothionein1M) axis [22]. Moreover, it acts as a tumor suppressor that modulates certain cellular functions such as proliferative activity, programmed cell death, motility, chemoresistance, and tumorigenesis across multiple human oncological conditions [23,24,25].

Although the role of *LINC00261* is explored in various cancers, comprehensive knowledge of its clinical potential has yet to be studied in hepatocellular, pancreatic, colorectal, and gallbladder cancers. Therefore, this review focused on how *LINC00261* mechanistically regulates the oncogenesis process in the cancers mentioned above and how it can be utilized as diagnostic and prognostic biomarkers of PC, CRC, HCC, and GBC. Furthermore, we give insight into how *LINC00261* can sponge multiple miRNAs to its target and regulate various cancer-associated signaling pathways.

## 2. Literature Search Methodology

The clinical and functional importance of *LINC00261*, a long non-coding RNA (lncRNA) linked to cancer biology, is examined in a recent study. The broad term “cancer” was combined with the key term “*LINC00261*” in a literature search on PubMed, Web of Science, Science Direct, DOAJ (Directory of Open Access Journals), and Google Scholar to find relevant articles. The review emphasizes important elements, such as lncRNA *LINC00261* expression dysregulation in various cancers, tissue samples, cell lines, and in vivo mice models, experimental procedures employed, target genes/proteins, and clinicopathological associations. Through in-depth analysis, we sorted 18 papers dated January 2025 that directly showed pertinence with *LINC00261* expression in various cancers, including pancreatic cancer (2020–2024), colorectal cancer, hepatocellular carcinoma, and gallbladder cancer. Two authors (S.D. and S.B.), under the direction of senior author A.J., improved the review’s depth even further. Through discussions and comprehensive analysis, the team confirmed the possible function of *LINC00261* among the functional characteristics of these cancers, including invasion, cell viability, and apoptotic activity.

## 3. Role of *LINC00261* in Various Cancers

The *LINC00261* is found to be aberrantly expressed in various cancers such pancreatic, gastric, colorectal, lung, hepatocellular, breast, laryngeal, endometrial, esophageal, prostate, choriocarcinoma, thyroid, and bile duct cancers [23]. Given the fact that there are specific biological characteristics of *LINC00261*, we have discussed the effects of the *LINC00261* and mechanisms governed by it in the tumorigenesis of pancreatic, colorectal, hepatocellular, and gallbladder cancers.

### 3.1. LINC00261 and Pancreatic Cancer

Multiple studies have indicated the critical involvement of diverse lncRNAs in the progression and treatment of pancreatic cancer (PC). For example, *HOTAIR* (*HOX transcript antisense intergenic RNA*), *MALAT1* (*Metastasis-associated lung adenocarcinoma transcript 1*), *HOTTIP* (*HOXA transcript at the distal tip*), and *PVT1* (*Plasmacytoma Variant Translocation 1*) have recently been found to be essential regulators linked to the progression of PC and have diagnostic and prognostic implications [26,27,28].

In one of the recent studies, *LINC00261* was investigated as an important regulator of PC, where authors have demonstrated that it is abnormally expressed in PC patients and cell lines compared to corresponding control samples. Most of these studies reported lower expression of *LINC00261* in PC, which is associated with its tumorigenesis [29,30].

*LINC00261* expression is significantly downregulated in pancreatic cancer (PC) cell lines, including AsPC-1 (Ascites Pancreatic Cancer-1), BxPC-3 (Biopsy xenograft of Pancreatic Carcinoma line-3), PANC-1 (Pancreatic Cancer-1), and CFAC-1 (cystic fibrosis pancreatic adenocarcinoma cell line), compared to normal pancreatic ductal epithelial cells (HPDE6-C7). The observed reduction, with a fold change of (~1.20), suggests a potential tumor-suppressive role in PC [31]. Moreover, through the TCGA (The Cancer Genome Atlas) database, the authors found low expression of *LINC00261* in PC patients compared to healthy individuals, which also correlates with poor patient survival [31]. This dysregulation may contribute to PC pathogenesis, indicating the need for further investigation into its functional and mechanistic implications.

To know how *LINC00261* affects the cell’s viability, invasion, and apoptotic activity in PC cells, the authors transfected the *LINC00261* overexpression vector into two PC cell lines named CFPAC-1 and BxPC-3 (Biopsy xenograft of Pancreatic Carcinoma line-3). Through the Western blot analysis, they found suppression of epithelial-mesenchymal transition (EMT), as evidenced by decreased expression of mesenchymal markers (N-cadherin, vimentin, MMP2) and a concurrent increase in epithelial marker E-cadherin, reinforcing cell-cell adhesion and limiting metastatic potential. These data suggest that an increase in the expression of *LINC00261* leads to less aggressive tumor phenotypes, better clinical outcomes, and improved prognosis [31]. These features of *LINC00261* suggest that it plays an important role as a tumor suppressor and can be utilized for future cancer-targeting strategies in PC. Moreover, through flow cytometry and transwell assays, it was found that *LINC00261* also plays a crucial role in inhibiting metastasis and increasing apoptosis. To further know how this *LINC00261* mechanistically suppresses (PC), *LINC00261*-targeted miRNA analysis was performed. For this, authors selected five miRNAs, namely miR-23a-3p, miR-21, miR-222, miR-193b, and miR-221, from the LinkedOmics TCGA database. Following an in-depth analysis using starBase v2.0 (https://rnasysu.com/encori/index.php), accessed on 4 December 2024 (an online bioinformatics tool), they found that miR-23a-3p exhibited stronger and more effective binding sequences with *LINC00261*. TCGA database analysis demonstrated a statistically significant inverse correlation (*p* < 0.0001) between *LINC00261* and miR-23a-3p, suggesting a functional axis that may contribute to PC progression. This highlights the emerging concept of lncRNA-miRNA interactions as critical regulatory networks in oncogenesis, providing a novel avenue for targeted therapy [31]. RNA Immunoprecipitation assays and dual-luciferase reporters further validate the direct interaction between *LINC00261* and miR-23a-3p [31]. Besides identifying in the plasma of patients with PC, Humeau et al. (2015) discovered that miR-23a acts as oncogenic and overexpressed in the saliva of PC patients with precursor lesions [32]. To further check the viability, PC cells BxPC-3 and CFPAC-1 were given a transfection containing several vectors, specifically with a vector + miR-con (control), a *LINC00261* overexpression vector + miR-con, or a *LINC00261* overexpression vector + miR-23a-3p and were assessed using the MTT (3-(4,5-Dimethylthiazol-2-yl)-2,5-diphenyltetrazolium bromide assay) test and found that miR-23a-3p partially reversed cell viability of these effects by lowering viability reduction and invasion reduction. Thus, *LINC00261* may inhibit PC progression by regulating miR-23a-3p and can be considered a possible target for PC therapy. These findings indicate that the *LINC00261*/miR-23a-3p axis could be explored as a potential therapeutic target, highlighting the importance of RNA-based interventions in cancer treatment. However, further research is still needed to better understand its role as a tumor suppressor molecule. Further mechanistic analysis revealed that *LINC00261* exerts its effects through post-transcriptional regulation via miRNA interactions. Bioinformatic screening using the LinkedOmics TCGA database and starBase identified miR-23a-3p as the most likely downstream target of *LINC00261*, with a statistically significant inverse correlation (*p* < 0.0001). Functional assays, including RNA immunoprecipitation and dual-luciferase reporter assays, confirmed direct *LINC00261*-miR-23a-3p binding, supporting a regulatory axis in PC pathogenesis. Given that miR-23a-3p has been previously implicated in oncogenic processes and detected in the plasma and saliva of PC patients, its interaction with *LINC00261* provides a compelling avenue for further therapeutic exploration.

Functional rescue experiments using MTT assays demonstrated that miR-23a-3p overexpression partially reversed the tumor-suppressive effects of *LINC00261*, restoring cell viability and invasion potential. This suggests that *LINC00261* inhibits PC progression by acting as a miRNA sponge, sequestering oncogenic miR-23a-3p to prevent downstream pro-tumorigenic effects. These findings highlight the *LINC00261*/miR-23a-3p axis as a promising therapeutic target; further in vivo validation and mechanistic insights are required to characterize its clinical relevance fully. Understanding the broader implications of *LINC00261*–miRNA interactions in pancreatic tumorigenesis could open new frontiers in RNA-based interventions for PC management.

In another study, the authors investigated the expression levels of *LINC00261* in 54 PCs and 54 neighboring non-cancerous tissues and found considerable downregulation (fold change = 2.3) [33]. This was confirmed by using GEPIA (Gene Expression Profiling Interactive Analysis) bioinformatics analysis, which also showed reduced *LINC00261* expression in PC tissues (*n* = 179) against normal tissues (*n* = 171) [33]. The authors used Kaplan–Meier survival curves to learn more about its clinical relevance, revealing that decreased *LINC00261* expression is associated with poor prognosis and adverse clinical features, such as greater tumor size, advanced TNM (Tumor, Node, Metastasis) stage, and higher metastatic rates [31,33].

**Figure 1:**
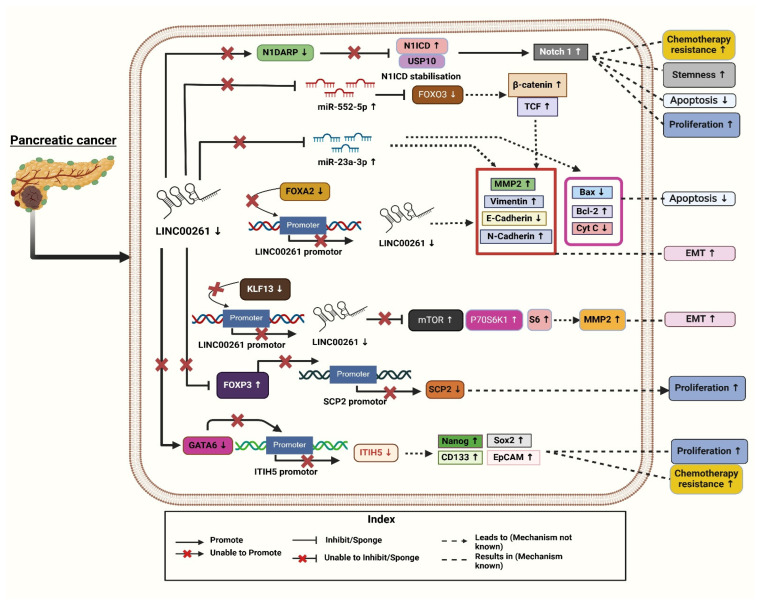
Molecular mechanisms associated with downregulation of *LINC00261* leading to the clinical progression and pathogenesis of pancreatic cancer. [Note: ↑—Upregulated, ↓—downregulated]. Created with BioRender.com.

Furthermore, it showed that the levels of *LINC00261* expression were significantly decreased in the PC cell lines MIA-PaCa2 (Mouse Insulinoma-Associated Pancreatic Cancer-2), Capan-2 (Carcinoma of the Pancreas-2), BXPC-3, PANC-1, CFPAC-1, and AsPC-1 in comparison with the human pancreatic epithelial cells. To further understand its function in vivo, it was discovered that mice injected with PC cells overexpressing *LINC00261* had greater survival rates and fewer metastatic foci than mice treated with control cells. In PC cells, *LINC00261* mainly targets the molecular level of miR-552-5p. These results from their study highlight the function of *LINC00261* in lowering PC metastasis [33]. Additionally, through TargetScan, the authors demonstrated that miR-552-5p has binding sites for *LINC00261*, which have been further validated using dual luciferase reporter assays. It was observed that the luciferase activity of *LINC00261* diminished in PANC-1 and MIA-PaCa2 cells co-transfected with the miR-552-5p and *LINC00261*-WT plasmid compared to miR-552-5p negative control in pancreatic epithelial cell lines [33]. Also, miR-552-5p was shown to target *FOXO3* (*Forkhead box O3*). *FOXO3* is a tumor suppressor and crucial regulator of the Wnt/β-catenin signaling pathway. It has been identified as a direct target of miR-552-5p [33]. Since it has been demonstrated that *FOXO3* regulates the Wnt signaling pathway, the expression of β-catenin and transcription factor 4 (TCF4) was assessed. *LINC00261,* by sponging miR-552-5p, restores *FOXO3* expression and suppresses the Wnt/β-catenin pathway, as evidenced by reduced levels of β-catenin and transcription factor 4 (TCF4). This suppression effectively inhibits epithelial–mesenchymal transition (EMT) and metastasis—key processes driving pancreatic cancer (PC) progression via the Wnt/β-catenin pathway. Conversely, reactivation of Wnt signaling leads to increased β-catenin and TCF4 expression, enhancing EMT markers and underscoring the critical role of the *LINC00261*/miR-552-5p/*FOXO3* axis in PC metastasis (Figure 1). However, these effects are reversed when miR-552-5p is reintroduced into *LINC00261* overexpressing PC cells [33]. Given the pivotal role of Wnt signaling activation in PC metastasis, *LINC00261*’s ability to restore *FOXO3* expression and suppress this pathway highlights its broader tumor-suppressive potential. This aligns with existing research emphasizing *FOXO3* as a key target in inhibiting pancreatic tumor progression. The *LINC00261*/*FOXO3* axis effectively disrupts EMT and metastasis, reinforcing its therapeutic significance [33].

Four molecular subtypes of pancreatic ductal adenocarcinoma (PDAC) were reported using the International Cancer Genome Consortium’s (ICGC) PDAC dataset: aberrantly differentiated endocrine exocrine (ADEX), immunogenic, pancreatic progenitor, and squamous. In this study, *LINC00261* was identified as the most significantly differentially expressed lncRNAs, with a substantial downregulation reported in the squamous subtype compared to the other three [4]. Further, to investigate the pathways linked to deregulated *LINC00261* expression in PDAC samples, the authors used gene set enrichment analysis (GSEA). They identified Forkhead box A2 (*FOXA2*), a chromosomal neighbor of *LINC00261*, as a direct regulator, with a strong association of (r = 0.72–0.91) across datasets and found a strong positive correlation between *LINC00261* and *FOXA2*, an epithelial marker and EMT inhibitor. Both *FOXA2* and *LINC00261* showed similar expression patterns across PDAC subtypes. To investigate the regulatory relationship, the authors altered *FOXA2* levels in PANC-1 cells [4]. *FOXA2* knockdown reduced *LINC00261* transcript levels, while overexpression increased RNA expression and promoter activity (Figure 1). *FOXA2* binding to the *LINC00261* promoter was confirmed by ChIP-qPCR (Chromatin Immunoprecipitation followed by quantitative Polymerase Chain Reaction). Further, the authors analyzed datasets, such as CCLE (Cancer Cell Line Encyclopedia), PDAC samples (Pancreatic Ductal Adenocarcinoma), and lung adenocarcinoma (LUAD), demonstrating that *LINC00261* expression correlated positively with epithelial markers (e.g., *CDH1*, *KRT19*, *CLDN7* (*cadherin 1*, *cytokeratin 19*, *claudin 7*) and negatively with mesenchymal markers, highlighting its role in regulating differentiation and EMT processes [4].

Additionally, the researchers further studied the effect of *LINC00261* for checking cell migration and invasion in PC cells (PANC-1) and by correlating its expression with *CDH1* (*Cadherin1*) encoding E-cadherin, which is a key epithelial–mesenchymal transition (EMT) marker. Mechanistically, the depletion of *LINC00261* was linked to reduced expression of *CDH1* (E-cadherin), a crucial protein for maintaining cell–cell adhesion. This suggests that the loss of *LINC00261* compromises E-cadherin-mediated adhesion, thereby enhancing cell motility and invasiveness. These results are consistent with clinical data showing that low *LINC00261* expression is associated with more aggressive pancreatic cancer (PDAC) phenotypes and worse patient outcomes, underscoring its potential as both a biomarker and a therapeutic target in pancreatic adenocarcinoma (PDAC) [4].

Based on GSE16515 (Gene Expression Series number 16515) and GSE32676 dataset analyses, Li Zou et al. (2021) reported that *LINC00261* expression is markedly reduced in PC tissues compared to normal controls. Their study identified a positive association between *LINC00261* and *Inter-Alpha-Trypsin Inhibitor Heavy Chain 5* (*ITIH5*) expression using ChIPBase (Chromatin Immunoprecipitation Database). Additionally, predictions from lncMAP indicated that *LINC00261* might have *ITIH5* expression through the transcription factor GATA6 (GATA Binding Protein 6), suggesting an important regulatory function of *LINC00261* in the progression of PC (Figure 1) [34]. The authors further examined the fact that *LINC00261* upregulates *ITIH5* expression in PANC-1 cells and stem cells by recruiting the transcription factor GATA6 to a specific binding site (site two) on the *ITIH5* promoter. Through a dual-luciferase assay, the authors confirmed that the overexpression of *LINC00261* enhances *ITIH5* promoter activity while its silencing decreases it. Bioinformatics analyses identified two potential GATA6 binding sites on the promoter, and experimental validation confirmed site two as the functional site for GATA6 binding. ChIP-qPCR assays demonstrated that GATA6 binds effectively to site 2 in a *LINC00261*-dependent manner, and RIP assays (RNA Immunoprecipitation Assays) established a direct interaction between *LINC00261* and GATA6. Silencing GATA6 in *LINC00261*-overexpressing cells abrogated *ITIH5* upregulation, indicating that the effect of *LINC00261* is mediated through GATA6 [34]. These findings suggest that *LINC00261* facilitates *ITIH5* expression by acting as a scaffold for GATA6 recruitment, highlighting a potential regulatory axis that could be explored for therapeutic interventions in PC. Functional assays demonstrated that increased *LINC00261* expression leads to the suppression of key stem cell markers (*Nanog*, Oct4 (Octamer-binding Transcription Factor 4), Sox2 (SRY-Box Transcription Factor 2), CD133 (Cluster of Differentiation 133), and EpCAM (Epithelial Cell Adhesion Molecule) and reduced cell viability, self-renewal capacity, invasive ability, and tumorigenicity. Additionally, it was observed that PANC-1 stem cells, which had restored *LINC00261* expression, exhibited reduced resistance to gemcitabine, the standard chemotherapy drug for PC. The overexpression of *LINC00261* in PANC-1 stem cells suggested its potential role as a tumor suppressor in PC. Various assays including sphere formation assays, transwell invasion tests, cytotoxicity assays, and tumor xenograft studies confirmed these effects. This indicates that upregulating *LINC00261* could help suppress the aggressive and drug-resistant characteristics of pancreatic cancer stem cells, providing a novel therapeutic approach to combat PC progression and improve drug sensitivity [34]. Moreover, flow cytometry analysis was conducted to check how it regulates the cell cycle, which revealed that overexpression of *LINC00261* increased the proportion of cells in the G0/G1 phase, suggesting an interruption in the cell cycle.

To gain more in-depth knowledge, researchers uncovered that *LINC00261* regulates PC progression through its interaction with FOXP3 (Forkhead Box Protein P3). FISH (Fluorescence in situ hybridization) assays revealed nuclear localization of *LINC00261* and FOXP3 in PC cells, while RIP and ChIP assays confirmed that *LINC00261* binds FOXP3, which in turn binds the SCP2 (Sterol Carrier Protein 2) promoter. Overexpression of *LINC00261* increased SCP2 expression while reducing FOXP3 levels, disrupting FOXP3-mediated suppression of SCP2. These findings suggest that the *LINC00261*/FOXP3/SCP2 axis influences PC development, presenting a novel regulatory mechanism and therapeutic target (Figure 1) [29].

Through GEO (Gene Expression Omnibus) datasets (GSE15471, GSE16515, and GSE32676), *LINC00261* was identified as significantly downregulated in PC tissues, with this reduction correlating to poor patient prognosis (*p* = 0.014). Survival analysis confirmed that patients with low *LINC00261* expression had decreased survival rates [15]. Functional assays in human PC cell lines revealed lower *LINC00261* levels in AsPC-1, Patu8988, MIAPaCa-2, and SW1990 cells than in normal pancreatic cells (HPDE6-C7). Additionally, RNA FISH showed that *LINC00261* was localized to both the nucleus and cytoplasm in PANC-1 cells, with a primary concentration in the nucleus. The authors further conclude that low *LINC00261* expression is a potential prognostic biomarker in PC, suggesting its tumor-suppressive role [15].

The authors investigated the potential of *LINC00261* as a prognostic biomarker for pancreatic cancer (PC). Initially, they transfected SW1990 cells with the pcDNA3.1-H-*LINC00261* plasmid. This process resulted in a significant increase in the levels of *LINC00261*. Additionally, to silence *LINC00261* in PANC-1 cells, the authors utilized the pLV3ltr-Puro-U6-*LINC00261*-i plasmid. This approach led to a reduction in *LINC00261* expression by up to 64.2%. It revealed that through transwell assays, *LINC00261* expression had no significant effect on cell growth, proliferation, or overall apoptosis rates; overexpression markedly suppressed PC cells’ migratory and invasive capabilities. These findings highlight *LINC00261*’s critical role in limiting tumor aggressiveness in PC [15]. The regulatory role of *LINC00261* in EMT and its downstream effects were thoroughly investigated. To demonstrate how *LINC00261* regulates PC progression, the authors used qRT-PCR and Western blotting to validate changes in the expression of key EMT markers and transcription factors [15].

Additionally, the analysis of the GEPIA database revealed that the Twist1 (Twist-related protein1) transcription factor is highly expressed in PC tissues. This Twist1 is known to play a critical role in promoting EMT by activating key target genes such as MMP2 (Matrix Metalloproteinase 2) in cancers. It was observed that the downregulation of *LINC00261* resulted in increased Twist1 transcription, thereby enhancing EMT processes. The findings reveal *LINC00261* as a pivotal regulator in mitigating PC aggressiveness, making it a potential therapeutic target for inhibiting EMT and tumor metastasis in PC. Also, their study discovered that the *KLF13* transcription factor upregulates *LINC00261* transcription by binding to its promoter. Bioinformatic analyses identified *KLF13* as a potential regulator and experimental validation showed that *KLF13* co-localized with *LINC00261* in the nucleus of PANC-1 cells. Overexpression of *KLF13* increased *LINC00261* expression significantly, and luciferase assays confirmed that *KLF13* directly binds to the *LINC00261* promoter, enhancing its activity [15]. Further, *LINC00261* interacts through specific RNA sequences by acting as a decoy molecule to prevent transcription factors from binding to their target DNA, thus either inhibiting or activating gene expression. *LINC00261* interacts through specific RNA sequences by acting as a decoy molecule to prevent transcription factors from binding to their target DNA, thus either inhibiting or activating gene expression. Moreover, the study shows that *LINC00261* suppresses mTOR-P70S6K1-S6 (mechanistic Target of Rapamycin-p70 S6 Kinase 1-S6 ribosomal proteins), a signaling pathway activation through *KLF13* regulation, which is critical in suppressing PC metastasis. The PI3K/Akt/mTOR (Phosphoinositide 3-Kinase/Protein Kinase B/mechanistic Target of Rapamycin) pathway is known to promote cell migration and metastasis by phosphorylating P70S6K1, leading to actin filament rearrangement and upregulating MMP2 expression. Overexpression of *KLF13* suppressed the pathway and decreased metastasis by preventing the activation of mTOR, P70S6K1-S6. However, when *LINC00261* was silenced, this inhibitory effect of *KLF13* was reversed, highlighting that *LINC00261* plays a pivotal role in modulating this pathway to prevent PC metastasis (Figure 1) [15]. By suppressing mTOR and its downstream effectors, *LINC00261* appears to inhibit metastatic spread, offering a potential molecular target for therapeutic intervention. With the increasing focus on PI3K/Akt/mTOR inhibitors in cancer therapy, further exploring *LINC00261*-mediated regulation in this pathway could provide novel treatment strategies.

Zhai et al. (2023) identified the highest differential expression of *LINC00261* between PC and normal cancer through the Cancer Genome Atlas (TCGA) database. Additionally, the authors found that the *LINC00261* had more potential for coding probability than other proven translatable lncRNAs evaluated by the online tool CPC2 (Coding Potential Calculator 2). Further, the results obtained by ORF Finder (Open Reading Frames) showed that ORF2, ORF7, and ORF12 could be translated. Additionally, the CCK8 (Cell Counting Kit-8) experiment revealed that only ORF12 overexpression prevented the growth of PC cells, implying that these ORFs may have biological roles. Moreover, the authors found *LINC00261* as a key regulator of Notch1 (Notch homolog 1) signaling in PC by encoding the microprotein Notch1 degradation-associated regulatory polypeptide (N1DARP). In normal conditions, Notch1 signaling controls crucial cellular processes like differentiation and apoptosis by promoting the degradation of the Notch1 intracellular domain (N1ICD) [30].

Moreover, in PC, hyperactivation of this pathway occurs due to the stabilization of N1ICD, driving uncontrolled cell proliferation, stemness, and chemoresistance (Figure 1). N1DARP, encoded by *LINC00261*, acts as a tumor suppressor by disrupting the interaction between N1ICD and the deubiquitinase USP10 (Ubiquitin-Specific Protease 10), leading to polyubiquitination and proteasomal degradation of N1ICD confirmed by the Western blotting analysis in CAPAN1 transfected with a vector or varying concentrations of FLAG-tagged (Fluorescent Antigen) N1DARP plasmid, where overexpressed N1DARP inhibited Notch activation. This results in reduced Notch1 activity, thereby inhibiting tumor growth, stemness traits, and chemoresistance [30]. The authors also highlighted the therapeutic potential of targeting this disrupted interaction, with the stapled peptide SAH-mAH2-5 derived from N1DARP offering a promising strategy for treating Notch1-activated PC. The authors validated their findings using both preclinical and clinical samples. Preclinical models included pancreatic cancer organoids (PDAC-1, PDAC-2, PDAC-R) derived from patient samples and genetically engineered KPC (Kras/p53/Cre) and KPNC (Kras/p53/Notch/Cre) mice [30].

Overall, the results above show that *LINC00261* has a tumor-suppressive effect in PC, influencing key pathways such as EMT, mTOR, and Notch signaling as illustrated in Figure 1. *LINC00261*’s downregulation is associated with poor prognosis and aggressive tumor phenotypes. However, further research is needed to investigate it as a viable biomarker for early PC detection and/or treatment. Despite its potential as a biomarker and therapeutic target, further research is needed to fully understand its mechanisms and validate its clinical application in combating pancreatic cancer progression and improving treatment outcomes.

### 3.2. LINC00261 and Colorectal Cancer

Colorectal cancer (CRC) is recognized as one of the most prevalent and aggressive malignancies affecting the digestive system, characterized by/known for high morbidity and mortality rates. It continues to pose a significant global challenge [35,36,37]. Despite recent improvements in therapy, patients with metastatic CRC still have a poor 5-year survival rate, which is critically low globally (~10%) despite recent developments in treatment [36]. Studies have demonstrated that numerous long non-coding RNAs play crucial roles in regulating CRC-related pathways [38]. Moreover, a poor clinical prognosis and the onset of metastasis in CRC patients are strongly associated with increased expression levels of *MALAT1* (*Metastasis-Associated Lung Adenocarcinoma Transcript 1*), *HOTAIR* (*HOX transcript antisense intergenic RNA*), and *H19* (*H19 Imprinted Maternally Expressed Transcript*) [39]. According to a recent study, the lncRNAs *KCNQ1OT1* (*KCNQ1 overlapping transcript 1*) and *WT1-AS* (*Wilms tumor 1 antisense RNA*) have substantial interactions with most miRNAs in the ceRNA network (competing endogenous RNA), indicating that they play important roles in the development of CRC [39].

In one of the recent studies, Liu et al. (2020) evaluated the clinical and mechanistic role of *LINC00261* in CRC. They used RNA expression profiling from the TCGA dataset, which included 459 non-metastatic colorectal CRC samples and 87 metastatic CRC samples, to examine metastasis-specific mRNAs, miRNAs, and lncRNAs in CRC tissue. By comparing non-metastatic and metastatic tissues, the results confirmed 628 differentially expressed (DE) mRNAs, of which 354 were upregulated and 274 were downregulated, along with 25 aberrantly expressed miRNAs and 144 dysregulated lncRNAs. Out of 144 dysregulated, 3-lncRNA, such as *LINC00114* (*long intergenic non-protein coding RNA 114*), *LINC00261*, and *HOTAIR*, were found to be highly significant in CRC patients compared to normal tissue samples. Additionally, analysis of lncRNA expression and clinical features in CRC showed *LINC00261* had a positive correlation with overall survival (OS) (*p* = 0.044 and *p* = 0.0006, respectively), but *HOTAIR* had a negative correlation with OS (*p* = 0.012). Moreover, the authors observed that *LINC00261* expression varies throughout the different stages of colorectal cancer. They noted significant reductions in the expression of lncRNA in stages III and IV of CRC in comparison to stages I and II CRC. Furthermore, the authors did not identify any significant differences when comparing combined CRC tissues to normal colorectal tissues. Their findings emphasize the importance of these lncRNAs as biomarkers in improving CRC prognosis and informing treatment decisions [39].

In terms of *LINC00261*, it is found that it exhibits positive coexpression with mRNAs involved in important biological processes such as “GO:0042158~lipoprotein biosynthetic process”, (Gene Ontology (GO), “GO:0008152~metabolic process”, “Drug metabolism cytochrome P450 (pigment 450)”, and “Chemical carcinogenesis” [39]. The expression of individual P450 variants has been associated with colorectal cancer, where some of these P450s show increased levels. Notably, P450 components showed high expression of CYP51 or CYP2S1 (Cytochrome P450 2S1), which has been correlated with a poor prognosis, with CYP51 (cytochrome P450 14α-sterol demethylase) serving as an independent prognostic indicator (15897573). The findings indicate that *LINC00261* is downregulated in colorectal cancer (CRC) and actively influences tumorigenesis and key cancer pathways.

Xi et al. (2023) investigated whether *LINC00261* plays a role in CRC through the miRNAs-mRNA axis. For this, they took the *LINC00261*-miRNA-148a/WNT10b axis. They established that targeting the *LINC00261*-miRNA-148a/WNT10b axis could significantly affect CRC cell proliferation and apoptosis, highlighting its importance in tumor growth and programmed cell death. Further, they found that by regulating the miRNA-148a/WNT10b axis, *LINC00261* may impact the proliferation of SW480 CRC cells. Further studies reported that transfection of *LINC00261*-specific siRNAs in CRC cells significantly increased miR-148a and decreased WNT10b (wingless-type MMTV integration site family, member 10B) and β-catenin proteins. The WNT10b is a key molecule of the Wnt/β-catenin signaling pathway, while miR-148a is reported as an oncogenic miRNA in CRC. According to the above findings, *LINC00261* may influence the advancement of colon cancer by interfering with the miR-148a/WNT10b axis and disrupting Wnt/β-catenin signaling [37].

Moreover, the reduced expression of *LINC00261* significantly decreased cell viability, increased the apoptosis rate, and caused G1-phase cell cycle arrest in CRC cells. These effects also underscore *LINC00261*’s potential in CRC cancer treatment [37]. It was also observed that higher expression levels of *LINC00261* tend to improve overall survival compared to CRC patients with lower expression levels. *LINC00261* is a tumor suppressor in colon cancer, showing that its overexpression inhibits cell growth and migration, and deactivates the Wnt signaling pathway. Both in vitro and xenograft models confirmed *LINC00261*’s role in slowing tumor progression, suggesting it could be a promising therapeutic target for colon cancer. From the data above, we can conclude that *LINC00261* could be linked to CRC patients’ prognoses, indicating that it could be a useful biomarker for patients with colon cancer [37].

Wang et al. (2018) demonstrated that *LINC00261* levels markedly down-regulated in tissues and cell lines of colon cancer, with the lowest expression observed in Stage III compared to Stages I and II [35], which also suggests its prognostic value. Furthermore, the authors observed that *LINC00261* significantly decreased cisplatin resistance in colon cancer in vivo while enhancing the drug’s efficacy by reducing tumor volume and weight. Their research also utilized the SW480 colon cancer cell line to investigate the relationship between *LINC00261* and cisplatin resistance and revealed that the cisplatin-resistant SW480 cell line exhibited markedly lower *LINC00261* expression than drug-sensitive cells [35]. It was observed that when *LINC00261* expression was increased in CRC cells through expression vector-based methods, the expression of pro-apoptotic proteins such as BAX (BCL2-associated X protein), FAS (FS-7-associated surface antigen, Fas receptor), Bim (Bcl-2-interacting mediator of cell death), and cleaved caspase-3 increases, which in turn reduced cell viability and migration of CRC cells by increasing E-cadherin and reducing EMT proteins such as MMP2 and MMP9 (Matrix Metalloproteinase-9) [35]. Further, it also blocked β-catenin nuclear translocation, which suppressed Wnt/β-catenin signaling and its target genes such as Myc (Myelocytomatosis oncogene) and CCND1 (Cyclin D1). The in vivo studies further indicated that the overexpression of *LINC00261* effectively inhibited both the initiation and progression of colon cancer [35]. *LINC00261* also synergized with cisplatin in the reduction of tumor growth in mice, indicating its potential therapeutic value in overcoming drug resistance and tumor progression. This research underscores that *LINC00261* plays an essential role in cell proliferation, migration, and apoptosis and mediated drug resistance in colon cancer cells. Other studies have indicated that overexpressing *LINC00261* could reverse drug resistance, inhibit CRC cell migration and invasion, and identify it as a promising molecule for CRC diagnosis and prognosis. These data provide a solid foundation for *LINC00261*-based innovative treatment strategies for CRC [35].

**Figure 2:**
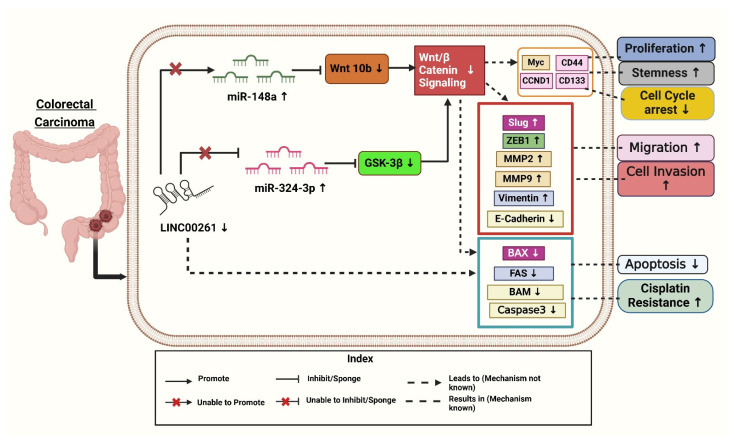
Downregulation of *LINC00261* and its effective targets leads to the progression of colorectal cancer. Created with BioRender.com [Note: ↑—Upregulated, ↓—downregulated].

Tang et al. (2022) identified an 8-lncRNA prognostic signature, including *SNHG7* (*small nucleolar RNA host gene 7*), *ZEB1-AS1* (*zinc finger E-box binding homeobox 1 antisense 1*), *U47924.27*, *NIFK-AS1* (*NIFK Antisense RNA 1*), *RP1-170O19.17*, *LINC00261*, *LINC00925*, and *CAPN10-AS1* (*Calpain 10 Antisense RNA 1*), as a potential independent prognostic factor for CRC patients based on the TCGA dataset. The predictive ability of this signature was validated using additional datasets (GSE39582, GSE29621 Gene Expression Omnibus Series) and effectively predicted chemotherapy responses [36]. Among these lncRNAs, *SNHG7*, *ZEB1-AS1*, *NIFK-AS1*, and others were positively associated with survival risk, whereas *LINC00261* was negatively correlated. In a recent study, *LINC00261* was significantly downregulated in CRC tissues and linked to lymph node metastasis and clinical stage (Figure 2) [40]. The signature identified high-risk patients with poor survival outcomes, as shown by Kaplan–Meier and ROC analyses (Receiver-operating characteristic curve), where the risk score became an independent prognostic factor. Immune profiling revealed differences in immune cell infiltration and mutational landscapes between risk groups, while IC50 analyses (Half-maximal inhibitory concentration) indicated varying sensitivities to chemotherapy drugs. Functional studies showed that lncRNAs, like *ZEB1-AS1* and *SNHG7,* inhibited CRC cells’ growth, migration, and EMT through PI3K/AKT signaling (phosphatidylinositol 3-kinase /protein kinase B), supported by Gene Ontology enrichment and pathway analyses. This indicates that *LINC00261* is a prognostic and therapeutic tool for CRC [40].

It was found that *LINC00261* expression was significantly reduced in (SW620, HT-29, DLD-1, HCT-116, and SW480) colon cancer cells compared to HCEPIC and normal colon cells [40]. In contrast, miR-324-3p levels were notably elevated in colon cancer cells. Further analysis showed overexpression of *LINC00261*, using a lentiviral vector (LV-*LINC00261*) in colon cancer cell lines HCT-116 and DLD-1, inhibits tumor progression by sponging the oncogenic miR-324-3p to activate GSK-3β (glycogen synthase kinase 3β), thus degrading β-catenin and inhibiting the Wnt/β-catenin pathway and resulting in decreased proliferation, migration, invasion, apoptosis enhancement, and decreased tumor growth both in vitro and in vivo. Overexpression of *LINC00261* reduced miR-324-3p levels and suppressed colon cancer cell proliferation, migration, and invasion, as demonstrated in HCT-116 and DLD-1 cell lines. Additionally, experiments confirmed that *LINC00261* upregulation inhibited cell proliferation [17].

Mechanistically, *LINC00261* repressed colon cancer progression by inactivating the Wnt signaling pathway by modulating miR-324-3p (Figure 2). These findings suggest that *LINC00261* reduces colon cancer by competitively binding itself to miR-324-3p and interfering with oncogenic signaling pathways [17]. As demonstrated in Figure 2, *LINC00261* is significantly downregulated in colon cancer, while miR-324-3p is upregulated. By inactivating the Wnt pathway and negatively regulating miR-324-3p, overexpression of *LINC00261* inhibits the spread of colon cancer and could serve as a target for therapeutic intervention [17].

### 3.3. Role of LINC00261 in Hepatocellular Cancer

Hepatocellular carcinoma (HCC) is a significant global health issue and is the fourth most prevalent cause of cancer-related deaths globally, ranking sixth in terms of incidence [8,19,41]. Numerous investigations have demonstrated that fatty acid metabolism is a vital metabolic process that supplies energy and signaling molecules, and encourages HCC [8].

**Figure 3:**
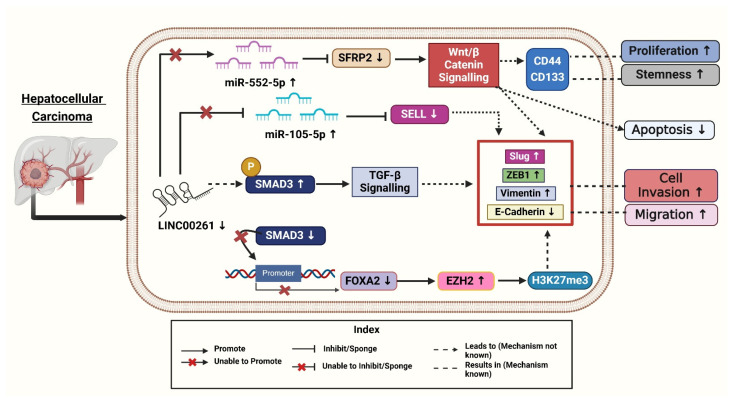
*LINC00261* and its downregulation affect various signalings ultimately leading to development of Hepatocellular cancers. Created with BioRender.com [Note: ↑—Upregulated, ↓—downregulated].

Chen et al. (2022) investigated the role of fatty acid (FA) metabolism-related lncRNAs in HCC. The authors aimed to identify key lncRNAs linked to FA metabolism and assess their prognostic and therapeutic potential. Using datasets from TCGA and Gene Expression Omnibus (GEO), they identified *LINC00261*, along with *SNHG1* (*Small Nucleolar RNA Host Gene 1*) and *SNHG7* (*Small Nucleolar RNA Host Gene 7*), as pivotal lncRNAs associated with FA metabolism [8]. To explore these associations, the study employed advanced bioinformatics techniques, such as single-sample gene set enrichment analysis (ssGSEA), to derive FA metabolism scores and correlate them with lncRNA expression. Experimental approaches, including qRT-PCR, FA metabolism PCR arrays, and Western blotting, were utilized to validate their findings and assess the downstream effects of these lncRNAs. Their results demonstrated that *LINC00261*, a key part of the FA metabolism-related lncRNA signature, plays a critical role in regulating FA metabolism, contributing to immune infiltration and epithelial-mesenchymal transition (EMT), processes linked to HCC progression and immune evasion. Patients with higher *LINC00261* expression exhibited better survival outcomes, underscoring its potential as a prognostic biomarker. Additionally, the study highlighted the significant molecular differences between patient subgroups based on FA metabolism-related lncRNA profiles, such as immune cell heterogeneity, genomic mutations, and EMT activity. These findings suggest that *LINC00261* holds promise as a therapeutic target and a predictor of immunotherapy response in HCC [8]. To get into clinical insights, the authors treated HCC cell lines Huh7 and HepG2 with Transforming growth factor-beta 1 (TGF-β1) to induce higher expression of EMT-associated proteins and observed reduced *LINC00261* expression [19]. To gain further insight, the authors investigated the clinical relevance of *LINC00261* in HCC and performed immunohistochemistry (IHC) on patient samples to correlate *LINC00261* expression and found that low levels of *LINC00261* were associated with high levels of p-SMAD3 (phospho-small mothers against decapentaplegic homolog 3), a molecule activated during TGF-β1 signaling, which promotes EMT (epithelial-mesenchymal transition), stemness, and metastasis (Figure 3). Moreover, it is observed that HCC patients having lower expression of *LINC00261* and a higher level of p-SMAD3 have overall poorer recurrence-free survival [19]. The above data suggest the combined potential of *LINC00261* and p-SMAD3 as useful biomarkers for predicting prognosis outcomes in HCC patients. To better understand *LINC00261*, the authors constructed *LINC00261* knockdown models using MHCC-LM3 (Hepatocellular Carcinoma cell line—Liver Metastatic Cell line 3) and SNU-449 (Seoul National University cell line 449) HCC cell lines. Through these models, they observed that migration and invasion were significantly promoted after *LINC00261* knockdown but were suppressed with *LINC00261* overexpression of the EMT-associated protein levels. The knockdown of *LINC00261* increased ZEB1 (Zinc-finger E-box binding homeobox 1) and vimentin levels while reducing E-cadherin expression, which indicated EMT promotion (Figure 3). Conversely, overexpression of *LINC00261* reversed these effects, decreasing ZEB1, Slug, and vimentin, while restoring E-cadherin levels. These findings highlighted that *LINC00261* suppressed EMT and limited cancer cell viability, suggesting its therapeutic potential in treating cancer by regulating molecular pathways involved in metastasis [19].

To uncover the mechanisms underlying *LINC00261*’s role in HCC, Chen et al. (2021) performed gain- and loss-of-function studies demonstrating that *LINC00261* suppresses migration, invasion, and epithelial-mesenchymal transition (EMT) in HCC cells [41]. Following this, the researchers investigated the molecular mechanisms involved, using advanced techniques like RNA pull-down assays to identify proteins interacting with *LINC00261*. The authors revealed that *LINC00261* recruits SMAD3 to the promoter region of FOXA2 (Forkhead Box A2), a neighboring tumor suppressor gene, thereby activating its expression [41]. Further bioinformatics analyses and rescue experiments confirmed that FOXA2 mediates the tumor-suppressive effects of *LINC00261* while restoring FOXA2 levels reversed the effects of *LINC00261* loss. To explore the silencing of *LINC00261*, the authors treated HCC cells with GSK126, an inhibitor of *Enhancer of Zeste Homolog 2* (*EZH2*) [41]. This treatment reduced the trimethylation of histone H3 at lysine 27 (H3K27me3) levels and restored *LINC00261* expression. Combining these findings with immunohistochemical staining of patient tissues, they linked high levels of *EZH2* and H3K27me3 to low expression of *LINC00261* and poorer patient outcomes (Figure 3). Overall, these experiments provided compelling evidence for the *EZH2*/*LINC00261*/FOXA2 axis as a critical regulator of HCC metastasis [41].

Ma et al. (2021) revealed that fucoidan is a natural polysaccharide derived from brown algae, exhibiting various biological activities, including antioxidant, anti-inflammatory, and antitumor properties. This compound has been shown to inhibit cell growth in vitro and in vivo, decrease invasion and motility, and trigger apoptosis and cell cycle arrest [42]. The antitumor efficacy of fucoidan has been validated in various cancers, including pancreatic, bladder, and ovarian cancers. Fucoidan inhibits tumor occurrence and development by modulating tumor immunity, obstructing angiogenesis, and disrupting cell cycle processes and apoptosis, indicating its significant potential in tumor therapy [42]. Fucoidan was found to upregulate *LINC00261*, which interacted with miR-522-3p and increased the expression of SFRP2 in HCC. Conversely, the down-regulation of *LINC00261* increases the expression of miR-522-3p and down-regulated SFRP2 expression and supports cell proliferation (Figure 3) [42]. This pathway is crucial in inducing dose-dependent arrest of the cell cycle and induction of apoptosis. It has been previously established that *LINC00261* acts as a tumor suppressor in terms of regulating the expression of miRNA. *LINC00261* could also be associated with reduced tumorigenicity of MHCC97H cells through miR-522-3p regulation in HCC. SFRP2 was significantly enhanced in the fucoidan treatment group, suggesting the interaction of *LINC00261*, miR-522-3p, and SFRP2 in HCC cells [42].

Song et al. (2022) investigated the role of the *LINC00261*/miR105-5p/SELL axis in HCC, particularly its connection to immune cell dysfunction and patient survival. This study highlighted *LINC00261* as a critical component of the *LINC00261*/miR105-5p/SELL axis, which influences immune cell function and patient survival in HCC [7], and explored how post-transcriptional regulatory mechanisms involving the *LINC00261*/miR105-5p/SELL axis influence immune dysfunction and overall survival in HCC patients. They utilized TCGA (The Cancer Genome Atlas) dataset samples (379 HCC out of these 337 tumor samples and 42 adjacent tissues) to perform comprehensive immune cell profiling and gene expression analysis with CIERSORT (Cell-type Identification by Estimating Relative Subsets of RNA Transcripts). Most of the patients were males with a median age of 61 and were diagnosed at an early stage of HCC as per the TNM staging system. In contrast to the surrounding tissues, tumor tissues exhibited considerably lower expression levels of SELL (L-selectin) (Log_2_ fold-change = −1.14) and *LINC00261* (Log_2_ fold-change = −0.90) [7].

In HCC tissues, the researchers found 201 differentially expressed miRNAs (DEMs), 216 differentially expressed lncRNAs (179 upregulated and 37 downregulated), and 3705 differentially expressed genes (1628 upregulated and 2077 downregulated). When compared to nearby normal tissues, tumor tissues showed downregulation of SELL (Selectin L). Selectin L is an important immunological regulator with adhesive properties, and its reduced expression contributes to cancer progression by promoting cell proliferation and metastasis [43]. Further, miR-105-5p plays a role in liver cancer (HCC) by influencing immune cells, especially B cells, through the *LINC00261*/miR-105-5p/SELL pathway. The presence of this miRNA has a direct association with the survival status of patients. Better overall survival (OS) was positively connected with its expression. When *LINC00261* attaches itself to miR-105-5p, it functions as a “molecular sponge”. Through this interaction, SELL expression is maintained by preventing miR105-5p from binding to the 3′ untranslated region (UTR) of the SELL mRNA. In tumor tissues, decreased *LINC00261* levels increase miR105-5p activation. Higher levels of *LINC00261* therapeutic target in HCC [7]. A more immunosuppressive tumor microenvironment (TME) results from improved immune cell homing and function caused by increased miR-105-5p suppressing SELL. By sponging miR105-5p, *LINC00261* functions as a competitive endogenous RNA (ceRNA), stopping miR105-5p from down-regulating SELL. In HCC samples, decreased *LINC00261* levels were accompanied by elevated miR105-5p levels, which inhibited SELL (Figure 3). Survival analysis showed higher SELL and *LINC00261* levels were linked to better results, whereas higher miR-105-5p predicted worse overall survival. Kaplan–Meier survival analyses confirmed SELL’s positive association with overall survival, further reinforcing its potential as a prognostic marker [7].

The above analysis reveals significant expression differences between tumor and normal tissues, identifying 3705 differentially expressed genes (DEGs) vital for cancer research. Upregulated DEGs are linked to cell division and DNA replication, emphasizing their role in tumor growth [7]. In contrast, downregulated DEGs are associated with immune responses, inflammation, and cell adhesion, suggesting potential therapeutic targets. The study found 201 differentially expressed miRNAs (DEMs) and 216 expressed long non-coding RNAs (DELs), enhancing our understanding of tumor biology and offering new research opportunities. *LINC00261* is significantly downregulated in HCC tissues, interfering with its function as a ceRNA (competing endogenous RNA) that protects SELL from miR-105-5p repression, which in turn leads to impairment of immunological functions and metastasis of HCC [7].

Furthermore, they established a ceRNA network identifying critical interactions influencing survival outcomes. Eleven immune cell types showed alterations in HCC tissues compared to non-cancerous tissues. The *LINC00261*/miR105-5p/SELL pathway emerged as a key marker, with decreased Selectin L, a molecule essential for immune cell homing and function, and was positively correlated with patient survival and markedly downregulated in HCC tissues [7]. This ceRNA axis implies restoring SELL levels may enhance immune function and outcomes in HCC. SELL expression correlates with improved overall survival. This underscores SELL’s importance in immune function and its connection to immunotherapy gene signatures, indicating potential for novel therapeutic approaches. The *LINC00261*/miR105-5p/SELL axis is a crucial prognostic biomarker and therapeutic target for HCC, substantially improving immunotherapy effectiveness. Further research is necessary to validate these findings and advance targeted treatments [7].

The above findings indicate that *LINC00261* plays a critical role as a tumor suppressor in HCC, and regulates various cancer-associated pathways, including the *EZH2*/FOXA2 and miRNA axes as shown in (Figure 3). The *LINC00261* also affects fatty acid metabolism, immune cell function, and the epithelial–mesenchymal transition. Targeting the mechanisms associated with *LINC00261* presents promising opportunities for enhancing immunotherapy and improving clinical outcomes in HCC patients. However, further research is needed to translate these findings into effective treatment

### 3.4. LINC00261 in Gallbladder Cancer

Although gallbladder cancer (GBC) is considered highly dangerous, only one study to date has examined the role of *LINC00261* in GBC. In that study, the researchers analyzed *LINC00261* expression in 100 paired clinical samples of GBC tissues and adjacent normal tissues using quantitative real-time PCR (qRT-PCR). Their findings revealed that patients with low *LINC00261* expression had significantly bad overall survival (OS, *p* = 0.0188) and progression-free survival (PFS, *p* = 0.0029) [44].

To elucidate the potential role of *LINC00261* in GBC, Niu et al. (2020) analyzed the relationship between *LINC00261* expression levels and the clinicopathological factors in GBC patients. Their findings indicated that lower levels of *LINC00261* expression were linked to a higher degree of differentiation (*p* = 0.017), an absence of liver metastases (*p* = 0.027), an advanced TNM stage (*p* = 0.008), and a bigger tumor size (*p* < 0.0001). The authors performed a Kaplan–Meier survival analysis to assess how *LINC00261* affects the prognosis of GBC patients. This analysis revealed that patients with low *LINC00261* expression had significantly poorer outcomes regarding overall survival (OS, *p* = 0.0188) and progression-free survival (PFS, *p* = 0.0029) [44]. Assessing *LINC00261* levels in tissue samples or serum could serve as a vital prognostic indicator for GBC, and incorporating this biomarker into clinical practice may improve patient outcomes and facilitate timely intervention strategies for GBC management [44]. Since the research on *LINC00261* in GC cancer is very scanty, the mechanistic role of *LINC00261* is still missing. Thus, more research is needed on these aspects.

## 4. Biological and Cellular Pathways Associated with *LINC00261*

**Figure 4:**
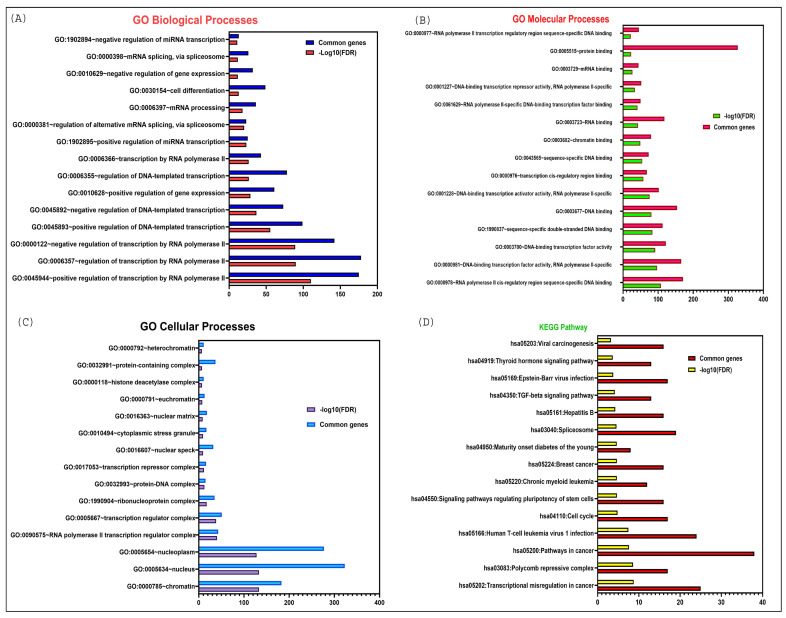
DAVID functional enrichment analysis of *LINC00261* target genes in various biological processes and signaling pathways. (**A**) Significantly enriched GO biological terms; (**B**) Significantly enriched GO molecular processes; (**C**) Significantly enriched GO cellular processes; (**D**) Significantly enriched KEGG pathway.

The targets of *LINC00261* play critical roles in various biological and cellular pathways. We conducted a Gene Set Enrichment Analysis of the targets to understand the contributions of *LINC00261* within different KEGG pathways and Gene Ontology (GO) biological, cellular, and molecular processes. As depicted in Figure 4A the results indicate that the target genes of *LINC00261* are primarily involved in significant biological processes, such as the regulation of miRNA and mRNA transcription, mRNA processing (including splicing), regulation of gene expression, and transcription by RNA polymerase II. This reinforces the antitumorigenic functions of *LINC00261* which were demonstrated through wet lab experiments of *LINC00261* binding to various miRNAs such as miR-550a-3p, miR-23a-3p, miR-148a, mir-324-3p, and miR105-5p [7,17,21,31,37].

In addition, these targets are actively engaged in various molecular functions, including protein binding, RNA binding (especially mRNA binding), DNA binding (such as chromatin binding and transcription cis-regulatory sequences), and other forms of sequence-specific DNA binding as demonstrated in Figure 4B. Moreover, they are involved in numerous cellular processes, including protein-DNA complex formation, ribonuclease complexes, histone deacetylase complexes, and the nuclear matrix, most of which are localized in the nucleus as elucidated in Figure 4C.

Finally, the KEGG pathway enrichment analysis revealed a significant association of *LINC00261* targets with cancer-related pathways, such as transcriptional misregulation in cancer, viral carcinogenesis, the TGF-beta signaling pathway, pathways regulating the pluripotency of stem cells, and the cell cycle Figure 4D.

## 5. Conclusions and Future Aspects

Cancer is an enormous health issue, impacting individuals and communities alike. This review highlights the essential role of *LINC00261* in influencing several biological functions, such as invasion, metastasis, apoptosis, angiogenesis, and proliferation. It has been identified as a tumor suppressor in gallbladder, hepatic, colorectal, and pancreatic cancers. According to the literature reviewed, the expression of *LINC00261* is found to be downregulated in PC, CRC, HCC, and GBC cancer tissues, as well as in cell lines (Table 1). This reduced expression is associated with worse outcomes, which correlates with poor prognosis and increased tumor growth. Lower levels of *LINC00261* lead to a decrease in results in epithelial markers, such as E-cadherin, while simultaneously increasing mesenchymal markers like vimentin and N-cadherin (Figure 1, Figure 2 and Figure 3). This shift from epithelium to mesenchymal tissue promotes tumor invasion and metastasis. Additionally, *LINC00261* interacts with specific miRNAs; for instance, it binds with miR-23a-3p and miR-552-5p in PC, miR-148a, and miR-324-3p in colorectal cancer, miR-522-3p and miR105-5p in hepatocellular carcinoma, to downregulate oncogenic pathways in these cancers [7,17,32,33,37,42].

This review discusses how *LINC00261* modulates oncogenic pathways in various cancers. Specifically, *LINC00261* suppresses the PI3K/Akt/mTOR (Phosphatidylinositol-3-kinase (PI3K), Protein kinase B (AKT), and Mammalian target of rapamycin (mTOR) signaling pathway by interacting with *KLF13* (*Krüppel-like factor 13*) in pancreatic cancer, thereby reducing metastasis and invasive characteristics. Additionally, the miR-148a/WNT10b axis inhibits tumor growth and enhances sensitivity to cisplatin when the expression levels of *LINC00261* are elevated through its interaction with WNT10b (wingless-type MMTV integration site family, member 10B) [37]. Furthermore, knocking down *LINC00261* results in increased cell proliferation due to the elevated levels of miR-522-3p in HCC. This increase in miR-522-3P subsequently reduces the expression of its downstream target, *SFRP2* (*Secreted Frizzled-Related Protein 2*) (Figure 3), which is critical for various cellular processes, including development, differentiation, and tumorigenesis [42].

It has been found that *LINC00261* contributes to transcriptional regulation by modulating transcription factors such as GATA6 (GATA binding protein 6), influencing the expression of *ITIH5* (*Inter-alpha-trypsin inhibitor heavy chain 5*), and encoding the microprotein N1DARP (Notch1 degradation-associated regulatory polypeptide). This microprotein disrupts hyperactive Notch1 signaling (Neurogenic locus notch homolog protein 1), which is a hallmark of PC progression, stemness, and chemoresistance. Overexpression of *LINC00261* has been shown to reduce cell motility, invasion, and chemical resistance, making it a promising option for targeted therapies. The multifaceted roles of *LINC00261* highlight its potential as a biomarker (diagnostic and prognostic) and in clinical treatment.

The prospects of *LINC00261* indicate great potential for transforming the clinical relevance of cancer diagnosis and treatment. The tumor-suppressive properties of *LINC00261* could serve as a foundation for future RNA-based therapies or microprotein-based approaches that aim to inhibit cancer development. Combining strategies targeting *LINC00261* with existing treatments could improve therapeutic outcomes by reducing metastasis, drug resistance, and tumor progression. Although its potential remains largely untapped, dysregulation of *LINC00261* has consistently been associated with poor patient outcomes, making it a reliable biomarker for diagnosis, prognosis, and therapeutic approaches. Large-scale clinical studies will be essential to validate its effectiveness as both a biomarker and a therapeutic target. Additionally, a deeper exploration of the regulatory networks surrounding *LINC00261* will facilitate its integration into precision medicine. By addressing critical factors driving cancer progression, *LINC00261* holds the promise to revolutionize the management of cancers such as pancreatic, colorectal, gallbladder, and hepatocellular carcinoma. However, despite the promising role of lncRNAs in cancer therapeutics, several limitations hinder their clinical application. One major challenge is the cellular penetration of long non-coding RNAs (lncRNAs), which requires improved stabilization against rapid degradation in the biological environment. To enhance their therapeutic potential, the development of optimized chemical structures is necessary to improve their stability and bioavailability [45].

Additionally, while lncRNAs are relatively easy to manufacture and can be chemically modified for enhanced protection against nucleotide degradation, their efficient delivery remains a significant hurdle. Liposomal delivery systems, which have been widely explored for lncRNA transport, face challenges such as scalability, reproducibility, chemical instability, and the risk of denaturation. Overcoming these barriers is crucial to harnessing the full therapeutic potential of lncRNAs, ensuring their safe and effective integration into precision oncology [45]

Overall, *LINC00261* represents a significant advancement in treating pancreatic, colorectal, hepatocellular, and gallbladder cancers. It provides a way for novel therapeutic and diagnostic approaches by altering important carcinogenic pathways. Further research is needed to validate the reported *LINC00261* findings in larger cohorts, explore its integration with existing therapies, and clarify its regulatory networks for therapeutic applications.

## Figures and Tables

**Table 1:** Clinical characteristics and properties of *LINC00261* in various cancers.

Cancer	Tissues/ Cell Lines Used	Approx. Mean Fold Change Compared to Normal	Property	Genes/Protein/ mi-RNAs Affected (Upregulation/Downregulation)	Biological Significance Upregulation/ Downregulation	Validation Method	Reference
Pancreatic cancer	CFPAC-1, BxPC-3, PANC-1, AsPC-1, MIA-PaCa-2, SW1990 Tissues	1.29 ↓ 2.3 ↓	Tumor suppressor	CDH1 ↓ CDH2 ↑ VIM ↑ FN1 ↑ N1ICD ↑ KLF13 ↓ mTOR ↑ SCP2 ↓ FOXP3 ↑ FOXA2 ↓ Nanog ↑ Oct4 ↑ Sox2 ↑ EpCAM ↑ CD CD133 ↑ β-catenin ↓ E-cadherin ↓ N-cadherin ↑ N1DARP ↓ miR-23a-3p ↑ miR-552-5p ↑	EMT ↑ Proliferation ↑ Invasion ↑ Cell viability ↑ Apoptosis ↓ Cell motility ↑ Metastasis ↑	qRT-PCR, RNA-FISH, CCK-8 assay, Transwell migration/invasion assays, Luciferase reporter assay, Western blot, RNA pull-down, RIP, ChIP, ELISA, Immunohistochemistry Flow cytometry, Matrigel-based tube formation assays, Microarray gene Cell sphere formation assay, MTT assay	[4,31,33]
Colorectal cancer	HCT116, HCT8, HT29, SW480, LOVO	2.17 ↓ 1.0 ↓	Tumor suppressor	β-catenin ↑ GSK-3β ↓ E-cadherin ↓ N-cadherin ↑ MMP9 ↑ WNT10b ↓ E-cadherin ↓ N-cadherin ↑ VIM ↑ ZEB1 ↑ miR-148a ↑ miR-135a ↑ mir-324-3p ↑	EMT↑ Proliferation ↑ Apoptosis ↓ Cell cycle arrest ↑ Migration ↑ Invasion ↑ Drug resistance ↑	qRT-PCR, flow cytometry, Western blotting, Transwell assay, CCK-8, Colony Formation Assays, Luciferase Assays, Immunohistochemistry (RIP), RNA pull-down assays, MTT assay	[17,37,39]
Hepatocellular carcinoma	HepG2, Huh7, MHCC-LM3, SNU-449	NA	Tumor suppressor	pSMAD3 ↑ VIM ↑ ZEB1 ↑ CD44 ↑ Slug ↑ FOXA2 ↓ EZH2 ↑ miR-522-3p ↑ SFRP2 ↓ miR105-5p ↑ SELL ↓	Cell adhesion ↑ Tumor Aggressiveness ↑ Cell Division ↑ Therapy Resistance ↑	qRT-PCR, Western blotting, Immunohistochemistry, Transwell assays, Tumor-sphere culture, RNA pull-down assays, FISH, Flow cytometry	[7,19,41]
Gallbladder cancer	SGC-996, GBC-SD, and NOZ	NA	Tumor suppressor	Not Studied	Tumor size ↑ TNM stage ↑ Degree of differentiation ↑	qRT-PCR	[44]

↑ Upregulation; ↓ downregulation; NA: not applicable. CDH1: Cadherin 1; VIM: vimentin; FN1: fibronectin; N1ICD: Notch1 intracellular domain; KLF13: Kruppel-like factor 13; SCP2: sterol carrier protein; FOXP3: Forkhead box P3; FOXA2: Forkhead box A2; Oct4: Octamer-binding; transcription factor 4; Sox2: SRY-Box Transcription Factor 2; EpCAM: Epithelial cell adhesion molecule; GSK-3β: Glycogen synthase kinase-3 beta; SMAD3: small mothers against decapentaplegic homolog 3; CD133: Cluster of Differentiation 133, SELL: Selectin L.

## Abbreviations

The following abbreviations are used in this manuscript:

PC	Pancreatic Cancer
CRC	Colorectal Cancer
HCC	Hepatocellular Cancer
GBC	Gallbladder Cancer
lncRNAs	Long non-coding RNAs
miRNAs	microRNA
GLOBOCAN	Global Cancer Observatory
ASR	Age-Standardized Rate
Ki-67	Antigen Kiel 67
TCONS_00027846	Transcript consensus number
DEANR1	Definitive Endoderm-Associated lncRNA1
ALIEN	A Long Intergenic Non-coding RNA enhancing Neuroblastoma
FALCOR	Folate Associated Long non-coding RNA
EBF1	Early B cell factor 1
BCSC	Breast Cancer Stem Cells
SDPR	Serum Deprivation Response Protein
EOC	Epithelial Ovarian Cancer
MT1M	Metallothionein1M
HOTAIR	HOX transcript antisense intergenic RNA
MALAT1	Metastasis-Associated Lung Adenocarcinoma Transcript 1
HOTTIP	HOXA transcript at the distal tip
PVT1	Plasmacytoma Variant Translocation 1
AsPC-1	Ascites Pancreatic Cancer-1
BxPC-3	Biopsy xenograft of Pancreatic Carcinoma line-3
PANC-1	Pancreatic Cancer-1
CFPAC-1	Cystic Fibrosis Pancreatic Adenocarcinoma Cell line
HPDE6-C7	Human Pancreatic Duct Epithelial cell line clone7
TCGA	The Cancer Genome Atlas
MMP2	Matrix Metalloproteinase 2
E-cadherin	Epithelial Cadherin
MTT	3-(4,5-Dimethylthiazol-2-yl)-2,5-diphenyltetrazolium bromide assay
qRT-PCR	Quantitative Reverse Transcription Polymerase Chain Reaction
GEPIA	Gene Expression Profiling Interactive Analysis
TNM	Tumor, Node, Metastasis
MIA-PaCa2	Mouse Insulinoma-Associated Pancreatic Cancer-2
Capan-2	Carcinoma of the Pancreas-2
FOXO3	Forkhead box O3
TCF4	Transcription Factor 4
EMT	Epithelial-Mesenchymal Transition
PDAC	Pancreatic Ductal Adenocarcinoma
ICGC	International Cancer Genome Consortium’s
ADEX	Aberrantly Differentiated Endocrine exocrine
FOXA2	Forkhead box A2
GSEA	Gene Set Enrichment Analysis
ChIP-qPCR	Chromatin Immunoprecipitation followed by quantitative Polymerase Chain Reaction
CCLE	Cancer Cell Line Encyclopedia
LUAD	Lung Adenocarcinoma
CLDN7	cadherin 1, cytokeratin 19, claudin 7
CDH1	Cadherin1
EMT	Epithelial-Mesenchymal Transition
GSE16515	Gene Expression Series number 16515
ITIH5	Inter-Alpha-Trypsin Inhibitor Heavy Chain 5
ChIPBase	Chromatin Immunoprecipitation Database
GATA6	GATA Binding Protein 6
Oct4	Octamer-binding Transcription Factor 4
Sox2	SRY-Box Transcription Factor 2
CD133	Cluster of Differentiation 133
EpCAM	Epithelial Cell Adhesion Molecule
FOXP3	Forkhead Box Protein P3
FISH	Fluorescence In Situ Hybridization
SCP2	Sterol Carrier Protein 2
GEO	Gene Expression Omnibus
Twist1	Twist-related protein1
mTOR-P70S6K1-S6	Mechanistic Target of Rapamycin-p70 S6 Kinase 1-S6 ribosomal proteins
PI3K/Akt/mTOR	Phosphoinositide 3-Kinase/Protein Kinase B/mechanistic Target of Rapamycin
TCGA	The Cancer Genome Atlas
CPC2	Coding Potential Calculator 2
ORF	Open Reading Frames
CCK8	Cell Counting Kit-8
Notch1	Notch homolog 1
N1ICD	Notch1 intracellular domain
USP10	Ubiquitin-Specific Protease 10
FLAG	Fluorescent Antigen
KPC	Kras/p53/Cre
KPNC	Kras/p53/Notch/Cre
KCNQ1OT1	KCNQ1 overlapping transcript 1
H19	H19 Imprinted Maternally Expressed Transcript
WT1-AS	Wilms tumor 1 antisense RNA
ceRNA	Competing Endogenous RNA
DE	Differentially Expressed
LINC00114	Long Intergenic Non-Protein Coding RNA 114
OS	Overall Survival
GO	Gene Ontology
P450	Pigment 450
CYP2S1	Cytochrome P450 2S1
CYP51	Cytochrome P450 14α-sterol Demethylase
WNT10b	Wingless-type MMTV integration site family, member 10B
BAX	BCL2-associated X protein
FAS	FS-7-associated surface antigen, Fas receptor
Bim	Bcl-2-interacting mediator of cell death
MMP9	Matrix Metalloproteinase-9
Myc	Myelocytomatosis oncogene
CCND1	Cyclin D1
SNHG7	Small Nucleolar RNA Host Gene 7
ZEB1-AS1	Zinc finger E-box binding homeobox 1 antisense 1
NIFK-AS1	NIFK Antisense RNA 1
CAPN10-AS1	Calpain 10 Antisense RNA 1
GSE39582	Gene Expression Omnibus Series
ROC	Receiver-operating characteristic curve
IC50	Half-maximal inhibitory concentration
PI3K/AKT	Phosphatidylinositol 3-kinase /Protein Kinase B
LV-*LINC00261*	Lentiviral Vector
GSK-3β	Glycogen Synthase Kinase 3β
SNHG1	Small Nucleolar RNA Host Gene 1
SNHG7	Small Nucleolar RNA Host Gene 7
ssGSEA	Single-Sample Gene Set Enrichment Analysis
TGF-β1	Transforming Growth Factor-Beta 1
IHC	Immunohistochemistry
p-SMAD3	Phospho-Small Mothers Against Decapentaplegic homolog 3
MHCC-LM3	Hepatocellular Carcinoma cell line—Liver Metastatic Cell line 3
SNU-449	Seoul National University cell line 449
ZEB1	Zinc-finger E-box binding homeobox 1
EZH2	Enhancer of Zeste Homolog 2
H3K27me3	Trimethylation of Histone H3 at lysine 27
CIERSORT	Cell-type Identification by Estimating Relative Subsets of RNA Transcripts
SELL	Selectin L
UTR	3′ Untranslated Region
TME	Tumor Microenvironment
ceRNA	Competitive Endogenous RNA
DEGs	Differentially Expressed Genes
DEMs	Differentially Expressed miRNAs
DELs	DNA Encoded Libraries
qRT-PCR	Quantitative Real-Time PCR
PFS	Progression-Free Survival
KLF13	Krüppel-like factor 13
SFRP2	Secreted Frizzled-Related Protein 2
N1DARP	Notch1 Degradation-Associated Regulatory Polypeptide
